# Hemocytes from *Pediculus humanus humanus* are hosts for human bacterial pathogens

**DOI:** 10.3389/fcimb.2014.00183

**Published:** 2015-01-30

**Authors:** Pierre-Julien Coulaud, Catherine Lepolard, Yassina Bechah, Jean-Michel Berenger, Didier Raoult, Eric Ghigo

**Affiliations:** CNRS UMR 7278, IRD198, INSERM U1095, Aix-Marseille UniversitéMarseille, France

**Keywords:** phagocytes, body lice, typhus

## Abstract

*Pediculus humanus humanus* is an human ectoparasite which represents a serious public health threat because it is vector for pathogenic bacteria. It is important to understand and identify where bacteria reside in human body lice to define new strategies to counterstroke the capacity of vectorization of the bacterial pathogens by body lice. It is known that phagocytes from vertebrates can be hosts or reservoirs for several microbes. Therefore, we wondered if *Pediculus humanus humanus* phagocytes could hide pathogens. In this study, we characterized the phagocytes from *Pediculus humanus humanus* and evaluated their contribution as hosts for human pathogens such as *Rickettsia prowazekii*, *Bartonella Quintana*, and *Acinetobacter baumannii*.

## Introduction

*Pediculus humanus humanus* is a strictly human ectoparasite with a worldwide distribution (Brouqui and Raoult, 2006) and represents a serious public health threat because it acts as a vector for pathogenic bacteria (Raoult and Roux, 1999). Human body lice may transmit epidemic typhus, which is caused by *Rickettsia prowazekii* (Bechah et al., 2008), the louse-borne relapsing fever, which is caused by *Borrelia recurrentis* (Houhamdi and Raoult, 2005), and trench fever, which is caused by *Bartonella quintana* (Badiaga and Brouqui, 2012). It has also been described that body lice can vectorize *Acinetobacter baumannii* (La Scola and Raoult, 2004). Because body lice are vectors of several human diseases, it is important to understand and identify the compartments (organs, tissue, cells) in which these bacteria reside to define new strategies to counterstroke the capacity of vectorization of the bacterial pathogens by body lice.

Whereas the immune systems of several invertebrates, such as mosquitos (Blandin and Levashina, 2007; Hillyer, 2009), shrimps (Tassanakajon et al., 2013), fruit flies (Kounatidis and Ligoxygakis, 2012), *Caenorhabditis elegans* (Pukkila-Worley and Ausubel, 2012), and more recently, *Mytilus galloprovincialis* (Koutsogiannaki et al., 2014), have been investigated, there is a crucial lack of knowledge concerning the immune system of body lice.

In 2012, evidence suggesting that the immune system of *Pediculus humanus humanus* relies on phagocytosis was reported (Kim et al., 2012), which implied the existence and function of phagocytic cells in these organisms. It is known that phagocytes from vertebrates can be hosts or reservoirs for several microbes. Therefore, we wondered if *Pediculus humanus humanus* phagocytes could hide pathogens.

In this study, we characterized the phagocytes from *Pediculus humanus humanus* and evaluated their contribution as hosts for human pathogens such as *Rickettsia prowazekii*, *Bartonella quintana* and *Acinetobacter baumannii*.

## Results

### Body lice hemocyte preparation and culture

**Figure 1:**
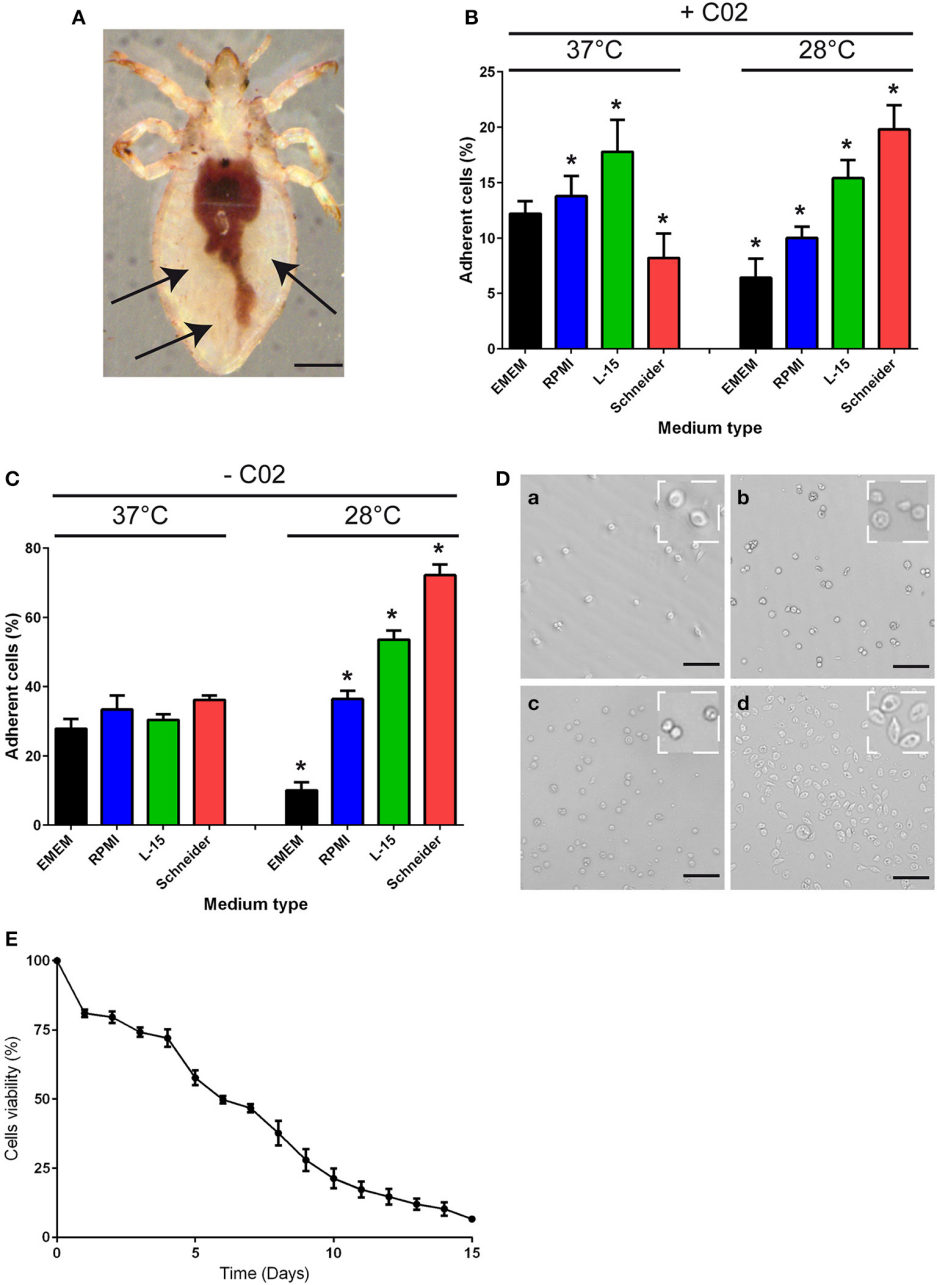
**Body lice hemocyte preparation and culture. (A)** Hemolymph was collected from the abdomen of *Pediculus humanus humanus* (black arrow) using an insulin syringe equipped with a 29G needle. Scale bar, 400 μm. **(B,C)** The collected hemolymph was added to various culture media in the **(B)** presence or **(C)** absence of CO_2_. After 16 h, the number of adherent cells in each condition was evaluated, and the percentage of adherent cells was calculated. The results are expressed as the means ± SDs (*n* = 5) (^*^p < 0.05). **(D)** Representative image of adherent cells observed under phase contrast microscopy after incubation at 28°C without CO_2_ in (a) EMEM, (b) RPMI, (c) L-15 medium, or (d) Schneider medium. Scale bar, 25 μm. **(E)** Hemocytes from *Pediculus humanus humanus* were cultivated in Schneider medium at 28°C without CO_2_ for 15 days, and their viability was evaluated each day by counting cells. The results are expressed as the mean percentages of viable cells ± SDs (*n* = 3).

To purify hemolymph phagocytes, we took advantage of the phagocytes' adherence to coated dishes. Hemolymphs in the abdomen of the body louse (Figure 1A) was collected and incubated at either 28 or 37°C in different culture media (EMEM, RPMI, L-15, Schneider) in the presence or absence of CO_2_ (Figures 1B–D), and the percentage of adherent cells was measured after 16 h of incubation. At 37°C or 28°C and in the presence of CO_2_, approximately 10% of cells were adherent, independent of the type of culture medium used (Figure 1B). Similar results were obtained at 37°C in the absence of CO_2_ (Figure 1C). At 28°C and in the absence of CO_2_, approximately 70% of cells were adherent when grown in Schneider medium (Figures 1C,D), whereas less than 55% of cells were adherent in the other medium conditions (Figures 1C,D). Furthermore, 7-day-old hemocytes could be maintained in Schneider medium without extensive cell death. Indeed, after 4 days of culture, cell viability of 72% was observed (Figure 1E), and after 7 days of culture, the cell viability decreased to 46%. Beyond 7 days, the cell viability decreased more rapidly, reaching 6% on the 15th day in culture (Figure 1E). Therefore, the subsequent experiments were performed in Schneider medium for no more than 7 days.

### Characterization of the phagocytic properties of body lice hemocytes

**Figure 2:**
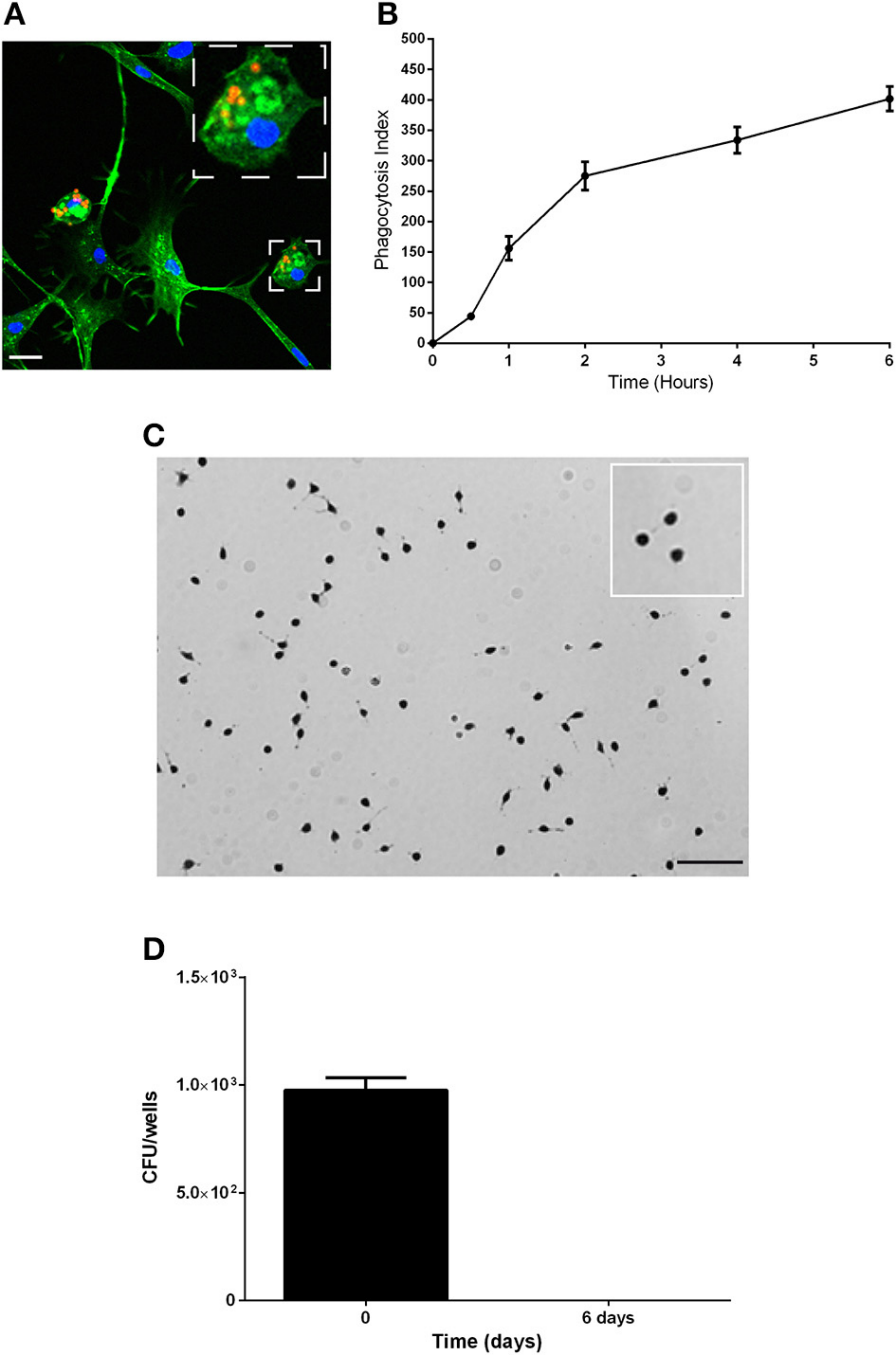
**Characterization of the phagocytic properties of the body lice hemocytes**. **(A,B)** The phagocytic capacity of the hemocytes was assessed based on their capacity to internalize latex beads (1/5000 dilution) over time at 28°C. **(A)** Representative image of actin-labeled cells (green) that had internalized latex beads (red). Scale bar, 10 μM. **(B)** The number of beads per cell and the percentage of cells containing engulfed beads were evaluated by microscopy, and these results were used to calculate the phagocytosis index. The mean ± SD is shown (*n* = 3). **(C)** The production of reactive oxygen species was evaluated using a NBT test. Cells were incubated with latex beads in Schneider medium at 28°C to stimulate the production of ROS, and the cells were observed by microscopy. Nearly more than 85% of cells were blue, indicating that they all produced ROS. Images representative of 3 experiments are shown. **(D)** The microbicidal activity of the hemocytes was evaluated by measuring their capacity to eliminate the non-pathogenic bacterial strain *E. coli* K12. Replication was evaluated by cfu counting. The results are shown as the means ± SDs (*n* = 2).

We then analyzed the functional properties of the isolated adherent cells to define their phagocytic and microbicidal activities. Mammalian cells that are able to ingest particles, generate ROS and clear bacteria are often considered phagocytes (Aderem and Underhill, 1999; Underhill and Ozinsky, 2002; Puertollano et al., 2011; Underhill and Goodridge, 2012). First, the capacity of the cells to phagocytose was evaluated (Figures 2A,B). The cells were incubated with latex beads at 28°C, and the number of beads captured per cell (Figure 2A) (phagocytosis index) was evaluated at various time points (Figure 2B). The adherent cells internalized latex beads in a time-dependent manner, and after 30 min, 17% of the cells had phagocytosed 2–3 latex beads; thus, the phagocytosis index was 44.2 ± 5.40 (Figure 2B). The percentage of cells that phagocytosed beads and the number of beads per cell increased over time. After 6 h, the phagocytosis index reached 401.6 ± 20.1, with 80% of cells having internalized at least 5 beads/cell (Figures 2A,B).

Second, the capacity of the isolated adherent cells to possess microbicidal activities was assessed by analyzing the ability of the adherent cells to produce ROS and to eliminate non-pathogenic bacteria. To evaluate ROS production, we used Nitro blue tetrazolium assays (NBT). We observed the formation of formazan precipitates in more than 85% of cells, which demonstrated that adherent cells produce ROS (Figure 2C). To evaluate the microbicidal capacity of the isolated hemocytes, cells were incubated with the non-pathogenic bacterial strain *E. coli* K12, and the behaviors of the bacteria were followed by cfu counting (Figure 2D). We found that *E. coli* were phagocyted by the hemocytes and then eliminated. Indeed, after 4 h (day 0) of incubation 976 ± 58 cfu were detected, and after 6 days, bacteria were not detected (no cfu). Taken together, these data show that hemocytes are able to phagocytose and that they have microbicidal activities; therefore, we named these adherent cells from the body louse hemolymph as body louse phagocytes (BLPs).

### BLPs are reservoirs for human pathogens

**Figure 3:**
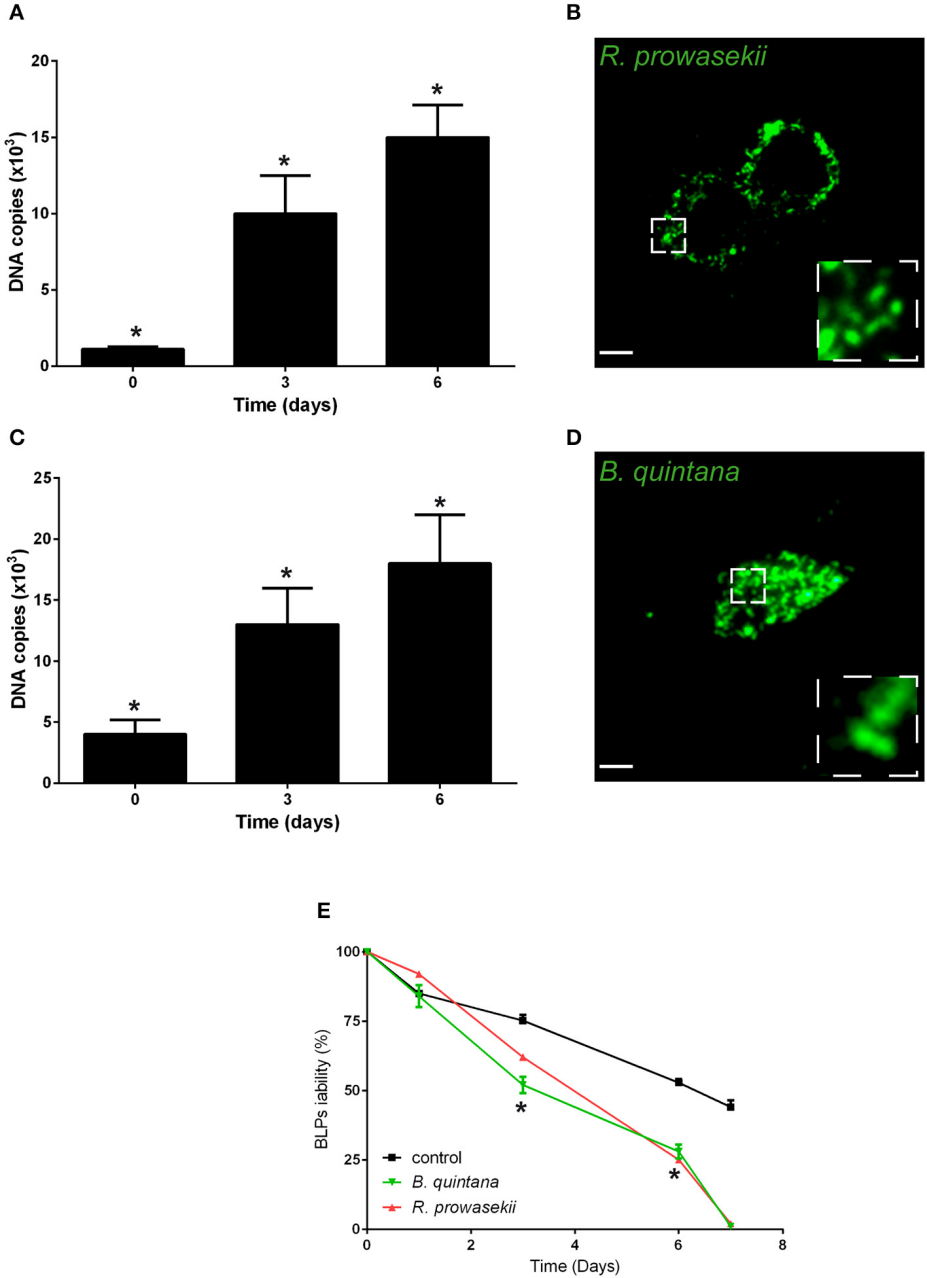
**BLPs are reservoirs for *R. prowazekii* and *B. quintana***. Body lice phagocytes were infected for 4 h (day 0) with *R. prowazekii* (100 bacteria-to-cell ratio) or *B. quintana* (100 bacteria-to-cell ratio), bacterial replication was then evaluated by real time PCR, and cell viability was evaluated. **(A)** *R. prowazekii* replicate in BLPs. The results are shown as the means ± SDs (*n* = 4, ^*^p < 0.05). **(B)** Representative epifluorescence microscopy image of body lice phagocytes infected with *R. prowazekii* (green) at 6 days post-infection. Scale bar, 25 μm. **(C)** *B. quintana* replicate in BLPs. The results are shown as the means ± SDs (*n* = 3, ^*^p < 0.05). **(D)** Representative epifluorescence microscopy image of body lice phagocytes infected with *B. quintana* (green) at 6 days post-infection. **(E)** Cell viability was evaluated by cell counting. The results are shown as the means ± SDs (*n* = 3, ^*^p < 0.05).

Next, we investigated whether BLPs may serve as hosts for bacterial pathogens. For that, we selected several microbes that are vectorized by *Pediculus humanus humanus*, including *R. prowazekii, B. quintana*, and *A. baumannii*. BLPs were infected with the set of selected microbes and cultivated for several days at 28°C in Schneider medium, and the bacterial behaviors and BLP viability were evaluated (Figure 3). After internalization, *R. prowazekii* survived and replicated in BLPs. Indeed, using real time PCR, we detected 1 × 10^3^ ± 140 copies of bacterial DNA after 4 h of infection (day 0); the number of copies of bacterial DNA increased at day 3 and then reached 1.5 × 10^4^ ± 2 × 10^3^ copies 6 days post-infection (Figures 3A,B). We observed that *R. prowazekii* replication dramatically affected the viability of the BLPs (Figure 3E), and thus bacterial replication led to BLP death and bacterial release into the culture medium. In a similar manner, we found that *B. quintana* was internalized by BLPs (4 × 10^3^ ± 1.20 × 10^3^ *B. quintana* DNA copies) and that *B. quintana* replicated in the phagocytes (1.8 × 10^4^ ± 4 × 10^3^ *B. quintana* DNA copies at day 6) (Figures 3C,D). As for *R. prowazekii*, BLPs infected with *B. quintana* exhibited decreased viability (Figure 3E). Surprisingly, we observed that *A. baumannii* was not internalized by BLPs, and this lack of internalization was independent of the infection time or the bacteria-to-cell ratio (Table 1).

**Table 1:** ***A. baumannii* is not internalized by BLPs**.

**Infection time (hours)**	***A. baumannii*-to-cell ratio**				
	**10**	**25**	**50**	**100**	**200**
2	0 cfu	0 cfu	0 cfu	0 cfu	0 cfu
4	0 cfu	0 cfu	0 cfu	0 cfu	0 cfu
6	0 cfu	0 cfu	0 cfu	0 cfu	0 cfu
12	0 cfu	0 cfu	0 cfu	0 cfu	0 cfu
24	0 cfu	0 cfu	0 cfu	0 cfu	0 cfu

*BLPs were incubated for different periods of time with various concentrations of A. baumannii, and A. baumannii uptake was evaluated by CFU counting*.

To complete the analysis, we compared the behaviors of the bacteria in BLPs to their behaviors in human macrophages. Interestingly, we found that *R. prowazekii*, *B. quintana*, and *A. baumannii* were able to infect human macrophages (Table 2). *R. prowasekii* and *B. quintana* survived but did not replicate in human macrophages, whereas *A. baumannii* replicated and induced the death of human macrophages (Table 2). Taken together, these results revealed that BLPs were unable to eliminate *R. prowazekii* and *B. quintana* and allowed their replication.

**Table 2:** ***R. prowasekii*, *B. quintana* or *A. baumanii* behaviors in human macrophages**.

	**Day 0**	**Day 6**	**Cell viability (%) at day 6**
*R. prowasekii* (DNA copy numbers)	1.6 × 10^5^ ± 1.2 × 10^3^	1.7 × 10^5^ ± 1.45 × 10^3^	83.6 ± 15.3
*B. quintana* (DNA copy numbers)	2.5 × 10^5^ ± 2.4 × 10^4^	3.4 10^5^ ± 2.8 × 10^4^	88.2 ± 13.8
*A. baumanii* (CFU)	1.0 × 10^4^ ± 2.0 × 10^2^	5 × 10^4^ ± 1.05 × 10^3^	5.4 ± 3.2

*Human macrophages were infected for 4 h (day 0) with R. prowazekii, B. quintana or A. baumannii, and bacterial replication was evaluated at day 6 by real time PCR or CFU counting*.

## Discussion

Several experimental models of body louse infestation (Houhamdi et al., 2002) have shown that body lice acquire *R. prowazekii* after feeding from an infected host, thereby allowing *R. prowazekii* to infect the epithelial cells of the upper gut of the lice (Houhamdi et al., 2002). While the immune systems of insects such as *Drosophila melanogaster* (Lemaitre and Hoffmann, 2007) have been carefully investigated, few studies have focused on the immune systems of body lice (Pedra et al., 2003; Kim et al., 2012). Recently, it was reported that the immune system of body lice involves a humoral immune response that requires phagocytosis (Kim et al., 2012). However, the cells involved in this process were not characterized, and their contributions to disease vectorization by the body louse *Pediculus humanus humanus* remained unknown. We have isolated hemocytes from body louse hemolymph and have unraveled the capacity of these cells to produce ROS and to internalize and eliminate non-pathogenic bacteria, similar to mammalian phagocytes. Thus, we suggest that body lice hemocytes are phagocytic cells (BLPs) that are fully equipped to have antimicrobicidal activity. Body lice, similar to other organisms, have an immune system containing phagocytes.

Next, we investigated the capacity of BLPs to be infected by human pathogens vectorized by *Pediculus humanus humanus*. BLPs were able to phagocytose pathogens such as *R. prowazekii* and *B. quintana*; however, despite their antimicrobial capacity, BLPs were unable to eliminate internalized *R. prowazekii*. In addition, we found that *R. prowazekii* and *B. quintana* replicated within BLPs. Interestingly, we observed that replication of *R. prowazekii* and *B. quintana* induced BLP lysis, and thus, the bacteria were released into the culture media. Surprisingly, *A. baumannii* is not internalized by BLPs, indicating that BLPs are not permissive to *A. baumannii*. We compared the behaviors of *R. prowazekii, B. quintana* and *A. baumannii* in BLPs to their behaviors in human macrophages. Interestingly, the becoming of *R. prowazekii* and *B. quintana* into human macrophages was different than in BLPs. Indeed, we observed survival of *R. prowazekii* and *B. quintana* in human macrophages; however, these bacteria replicated strongly in BLPs. This finding suggests that BLPs most likely do not have the microbicidal equipment to kill *R. prowazekii* and *B. quintana*, in contrast to human macrophages. Unlike *A. baumannii*, *R. prowazekii* and *B. quintana*, we found that *A. baumannii* were not internalized by BLPs, whereas there were internalized by macrophages. It is possible that receptors allowing *A. baumannii* uptake in mammalian phagocytes are not expressed by BLPs or are not conserved from mammalian phagocytes to BLPs.

Our results suggest that BLPs might host microbes and contribute to making *Pediculus humanus humanus* a vector for human diseases. In addition, our data provide new knowledge about the possible localization of human pathogens in body lice. It was known that *R. prowazekii* invades the gut cells (Houhamdi et al., 2002); here, we discover that hemocytes can also be hosts for this pathogenic bacterium. Moreover, the death of the BLPs during bacterial replication might contribute to the spreading of the bacteria into the body lice, and thus, this could be a method for bacterial contamination of the host. It is possible that some viruses and microbes that are responsible for human diseases have no identified vectors because there are hiding in hemocytes, which is small population of the cells of body lice (we scored ~750 hemocytes/body lice), and thus, the microbes responsible for human diseases could not be detectable using the classical methods of investigation. We also suggest that, in the near future, it will be important to search for viruses and microbes that infect BLPs because as amoebas, BLPs could be reservoirs for unidentified pathogens. In conclusion, we have characterized phagocytes of body lice and unraveled their capacity to be vectors for human pathogens.

## Materials and methods

### Media

PMI 1640, DMEM Leibovitz's 15 medium, and Schneider medium were obtained from Invitrogen and were supplemented with 10% fetal calf serum (Gibco-BRL) and 100 U/ml penicillin (100 U/ml), streptomycin (50 μg/ml), gentamycin (10 μg/ml), and vancomycin (5 μg/ml). Before the experiments, the antibiotics were removed by extensive washing.

### Bacterial strains

*Rickettsia prowazekii* (Rp22 strain) (Birg et al., 1999; Bechah et al., 2010), *Bartonella quintana* strain Oklahoma (ATCC 49793) (Kernif et al., 2014), and *Acinetobacter baumannii* homeless isolate (La Scola and Raoult, 2004) were grown as previously described.

### Body lice strains

Colonies of *Pediculus humanus humanus*, strain *Orlando*, were grown as previously described (Fournier et al., 2001).

### Hemolymph collection

*Pediculus humanus humanus* were starved for 48 h and then washed 3 times in each of four successive solutions: solution A, phosphate-buffered saline (PBS), pH 7, plus Tween 80 (0.1%); solution B, sterile water; solution C, 70% ethanol; solution D, sterile PBS, pH 7. The hemolymph was collected from the abdomens of body lice using an insulin syringe equipped with 29G needles. The collected hemolymph was added to the culture media.

### Cell viability

The percentage of adherent cells was measured using a phase contrast microscope (Leica DMI 3000 B; Leica, France) as previously described (Prescott and Breed, 1910).

### Phagocytosis assay

Cells were incubated at day 3 with latex beads (1 μm, Sigma) at 28°C, washed extensively to remove non-internalized beads and then fixed with 3% paraformaldehyde for 20 min. Using an epifluorescence microscope (Leica DMI 3000 B), the numbers of latex beads per cell and the numbers of cells containing latex beads were evaluated. The phagocytosis index is defined as (the average number of latex beads per cell in cells containing latex beads) × (the percentage of cells containing beads).

### Detection of reactive oxygen species

The production of reactive oxygen species was evaluated using the NBT test, as previously described (Jozefowski and Marcinkiewicz, 2010). The cells were incubated with latex beads (1 μm, Sigma) for 2 h in Schneider medium at 28°C to induce the production of ROS.

### Bacterial infection

BLPs were infected with *R. prowazekii*, *B. Quintana*, or *A. baumannii* and then extensively washed to remove the free bacteria; the BLPs were then incubated further. In some experiments, the bacteria were visualized by immunofluorescence, as previously described (Bechah et al., 2010), and cellular F-actin was stained using Alexa 488-conjugated phallacidin. Infection was quantified by real time PCR or cfu counting.

### Statistical analysis

The results are expressed as the means ± SDs and were analyzed using the nonparametric Mann–Whitney *U*-test. Differences were considered significant at *p* < 0.05.

### Conflict of interest statement

The authors declare that the research was conducted in the absence of any commercial or financial relationships that could be construed as a potential conflict of interest.
